# Multiscale characterization of commercial and landfill-recovered plastics in Songkhla province, Thailand

**DOI:** 10.1039/d5ra03620h

**Published:** 2025-07-11

**Authors:** Nisamon Thongham, Parinya Khongprom, Chontira Sangsubun

**Affiliations:** a Faculty of Science and Digital Innovation, Thaksin University Phatthalung 93210 Thailand schontira@tsu.ac.th; b Department of Chemical Engineering, Faculty of Engineering, Prince of Songkla University Songkhla 90112 Thailand kparinya@eng.psu.ac.th; c Air Pollution and Health Effect Research Centre, Prince of Songkla University Songkhla 90112 Thailand kparinya@eng.psu.ac.th

## Abstract

This study provides a comprehensive assessment of five types of plastics: commercial polyethylene (PE), polypropylene (PP), polyethylene terephthalate (PET), polyvinyl chloride (PVC), and landfill-derived polyethylene [PE (LF)] from Songkhla Province, Thailand. PE (LF) was naturally exposed to environmental conditions. Through advanced multiscale characterization techniques, including scanning electron microscopy (SEM), thermogravimetric analysis (TGA), tensile testing, and Fourier-Transform Infrared spectroscopy (FTIR), it was found that prolonged exposure to landfill conditions causes substantial physical, mechanical, and chemical degradation in PE (LF), as evidenced by extensive surface cracks, reduced tensile strength (below 10 MPa), and the formation of oxidative functional groups (C=O and –OH). In contrast, commercial plastics maintain superior stability and mechanical integrity. Despite this degradation, PE (LF) retains significant potential for energy recovery *via* pyrolysis, with thermal properties suggesting efficient conversion into hydrocarbon fuels. Our findings highlight the urgent need for innovative waste management strategies and position landfill-derived plastics as a valuable resource for sustainable energy solutions. This research offers actionable insights for waste management authorities and recycling industries, supporting the transition toward a circular economy and the reduction of environmental impact from plastic waste.

## Abstract 2

Landfill-recovered PE from Songkhla, Thailland, was analyzed to assess its thermal, chemical, and morphological properties. Results support its potential for pyrolysis-based energy recovery from post-consumer plastic waste.

## Graphical abstract

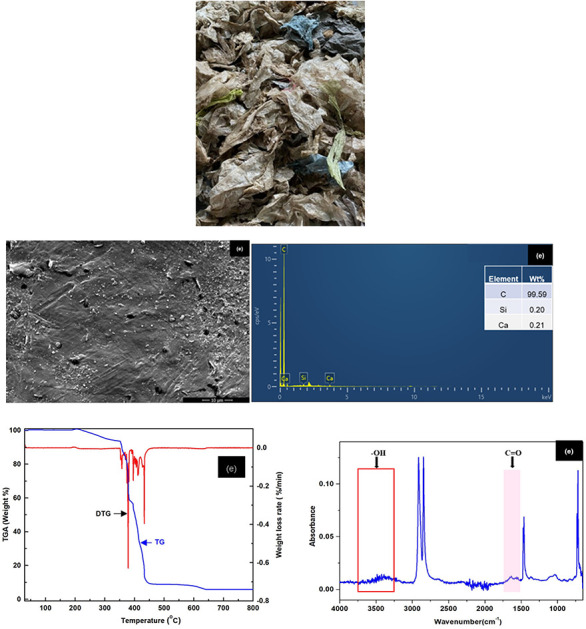

## 1. Introduction

The increasing volume of plastic waste is now one of the most important environmental challenges in the world. Urban centres, especially those with rapid population and industrial activity growth, are particularly vulnerable in waste management. Municipal landfills, originally designed for effective waste disposal, have been overwhelmed by non-biodegradable plastics, causing long-term environmental damage. Polyethylene (PE), polypropylene (PP), polyethylene terephthalate (PET), and polyvinyl chloride (PVC) contribute significantly to waste streams.^1–3^ These materials play an essential role in modern life, especially in packaging, construction and electronics, due to their chemical stability, flexibility and cost-effectiveness. However, the very longevity that makes these plastics advantageous also contributes to their longevity in the environment for centuries if they are not properly managed.^4,5^ A misdeposit of these plastics causes serious environmental degradation and contaminates both soil and water. For example, PET and PVC release harmful chemicals such as Bisphenol A (BPA) and Phthalates, which affect terrestrial and aquatic ecosystems. Furthermore, plastic accumulation in landfills increases greenhouse gases, such as methane, in decomposition, and aggravates climate change.^6,7^

In the context of rapid urbanization, the province of Songkhla faces unique challenges in the management of plastic waste due to limited landfill space and insufficient infrastructure for recycling and waste-to-energy initiatives. However, there are opportunities for innovative solutions. Researchers increasingly turn to waste-to-energy technologies such as pyrolysis, gasification, and incineration to convert plastic waste into fuel.^8–12^ These technologies not only reduce the volume of plastic waste in landfills, but also provide a renewable energy source, which is crucial for the search for alternatives to fossil fuels.

The chemical properties of the plastic studied are closely related to their potential for energy recovery. Each type of plastic—such as polyethylene (PE), polypropylene (PP), polyethylene terephthalate (PET), and polyvinyl chloride (PVC)—possesses a distinct chemical structure, which directly influences its physical and chemical characteristics, including thermal stability, melting point, and degradability. These properties are critical in determining the appropriate technology for energy conversion, such as pyrolysis or incineration.^13,14^ For example, PE and PP consist primarily of hydrocarbon chains with high carbon and hydrogen content, low density, and an absence of chlorine atoms. These features contribute to their high calorific values (exceeding 40 MJ kg^−1^), making them particularly suitable for conversion into liquid fuels or gaseous products *via* pyrolysis.^14,15^ In contrast, PET contains ester groups and oxygen atoms in its molecular structure, which result in higher melting points and enhanced thermal stability, thus requiring higher temperatures during pyrolysis.^13^ Although PVC also possesses a high calorific value, the presence of chlorine in its structure can lead to the release of toxic substances (*e.g.*, hydrogen chloride, HCl) during thermal processing, necessitating stringent emission control measures.^14^

Chemical degradation of plastics, especially long-term landfilled PE, involves oxidative reactions that introduce carbonyl (C=O) and hydroxyl (–OH) functional groups. These modifications reduce the structural integrity of the polymer, facilitating chain scission during pyrolysis. As a result, the energy required to initiate the reaction is lower, and hydrocarbon yields tend to increase.^15^ Additionally, analytical techniques such as FTIR and EDX confirm compositional changes and the presence of contaminants, both of which are relevant to the energy recovery potential.

Understanding the mechanical, physical, and thermal properties of plastics is essential to optimizing waste-to-energy technologies. Plastics such as PET, PE, PVC and PP fusion points, tensile strength, thermal stability and density determine the adaptability of various energy recovery methods.^16–20^ The unique properties of each plastic type have a significant impact on the behaviour under thermal and mechanical pressures. For example, PET has a melting point of about 280 °C,^21,22^ which is especially suitable for pyrolysis, and PE has lower melting points and greater flexibility, resulting in challenges in combustion processes. Thermal thermometry analysis (TGA) has shown that PET decomposes at about 300 °C, while PE and PP begin to decompose at about 350 °C, making them suitable for the recovery of high-temperature energy.^21,23^ Furthermore, PET density (1.34 g cm^−3^) contrasts with PE density (0.93 g cm^−3^), which affects processing technology. The detailed study of PET crystal structure by Daubeny *et al.* (1954)^24^ has provided fundamental insights into the relationship between polymer arrangement and thermal properties, supporting the understanding of PET's high melting point and thermal stability. Scan electron microscopes (SEMs) reveal different surface structures: PET has a solid and smooth surface, while PE is irregular and porous, which may improve decomposition and energy recovery during thermal processing. Differential thermogravimetric data (DTG) indicate that PE decomposes faster than PP and is potentially more efficient for energy release during thermal conversion.^25–27^ A detailed understanding of these properties is essential, as it serves as a basis for optimising the transformation processes while minimising the environmental impact of waste-energy technologies.

In recent years, testing methods for assessing the mechanical and physical behaviour of plastics have advanced considerably. Recent advances in polymer characterization, as demonstrated by Salakhov *et al.* (2021),^28^ have enhanced the precision and scope of mechanical and physical property assessments for various plastics, including the use of advanced analytical techniques and novel synthetic approaches. Early works by Rubiano-Navarrete *et al.*^29^ established basic protocols for tensile resistance tests, which are still essential to evaluate the integrity of plastics during thermal decomposition. At the same time, the development of analytical techniques such as scanning electron microscopes (SEM) and energy-dispersive X-ray spectroscopy (EDX) provides important insights into the surface shape and elements of plastic waste. These technologies, together with TGA and DTG analyses, continue to improve understanding of degradation behaviours and energy potentials, thus improving the efficiency of plastic fuel conversion systems. Furthermore, the environmental impact of long-term plastic degradation in landfill conditions has become a focus of recent research. Limited waste management capacity and increasing pressure on landfills emphasize the urgency of integrated solutions. They must combine technological innovations such as the application of waste to energy, with effective policy interventions such as stricter regulations on plastic production and disposal. Social and economic dimensions, including employment opportunities in the recycling sector and public awareness initiatives to reduce plastic consumption, are also vital to a holistic waste management strategy. As the world community is facing increased plastic pollution, it is clear that there is no single solution. A comprehensive approach, including progress in recycling technologies, material characterization improvements, sustainable energy recovery methods and multi-dimensional policy reforms, is essential to effectively mitigate the environmental impact of plastic waste.

This study compares the old-fashioned behaviour and properties of commercial plastics, including polyethylene (PE), polypropylene (PP), polyethylene terephthalate (PET) and polyvinyl chloride (PVC), with landfill polyethylene (PE) obtained from a local landfill in Songkhla province of Thailand. The study involves a comprehensive description through scanning electron microscopes and energy dispersion X-ray spectroscopy (SEM-EDX), thermogravimetric analysis and differential thermogravimetric (TGA/DTG), tension testing, and Fourier Transformation Infrared spectroscopy (FTIR). The data obtained from this comparison will provide valuable information on the differences in the physical, chemical, thermal and mechanical properties of commercially produced plastics and those exposed to long-term garbage disposal conditions. The conclusions will contribute to improving waste management practices and developing sustainable transportation. In addition, the environmental implications of long-term plastic degradation in landfill environments have become a centre of attention for recent research. The limited capacity for waste management and the increasing pressure on waste dumps highlight the urgency of integrated solutions. These must combine technological innovations, such as the use of waste in energy, with effective policy interventions, such as stricter plastic production and disposal regulations.

## 2. Materials and methods

### 2.1. Site specification

Songkhla is a coastal province in southern Thailand, consisting of multiple local administrative units. Among these, Hat Yai Municipality stands out as the largest and most urbanized, serving as the province's economic, commercial, and transportation hub. Covering an area of 21.00 km^2^, Hat Yai Municipality had an estimated population of approximately 191 696 in 2024, making it the fourth-largest city municipality in Thailand.^30^ Its proximity to the Malaysian border, rapid urbanization, and population growth have all contributed to significant municipal solid waste (MSW) generation.^31^

Hat Yai is the largest waste generator in Songkhla province, primarily due to its high population density, extensive commercial activity, vibrant tourism sector, and numerous educational institutions. The city produces approximately 237.75 tons of household waste per day.^32^ The major components of the municipal waste stream are consistent with those observed in other urban centers in Southeast Asia: organic waste—including food scraps and garden residues—accounts for the largest proportion, followed by plastic waste from commercial and household sources (such as packaging materials and single-use plastics), as well as glass and metal containers.^33^

Despite various waste reduction and recycling initiatives, landfilling remains the dominant method of waste disposal in Hat Yai. The city's sanitary landfill, established in 1999, is located in Tambon Khuan Lang, approximately 10 kilometers from the city center. Spanning about 100 rai (approximately 16 hectares or 39.5 acres), the site serves not only Hat Yai but also several nearby municipalities, including Ban Phru, Kho Hong, Ban Rai, and Phatong.^34^ Currently, the landfill remains a critical part of the city's waste management infrastructure, particularly for handling non-recyclable, non-combustible, and residual waste that cannot be processed by recycling or waste-to-energy systems. To mitigate odor, pest infestation, fire hazards, and environmental impacts, incoming waste is compacted and routinely covered with soil, which also helps reduce wind-blown litter and surface water infiltration. Leachate and landfill gas are managed using passive control systems, although the potential for improved technologies has been recognized.^34^

**Fig. 1:**
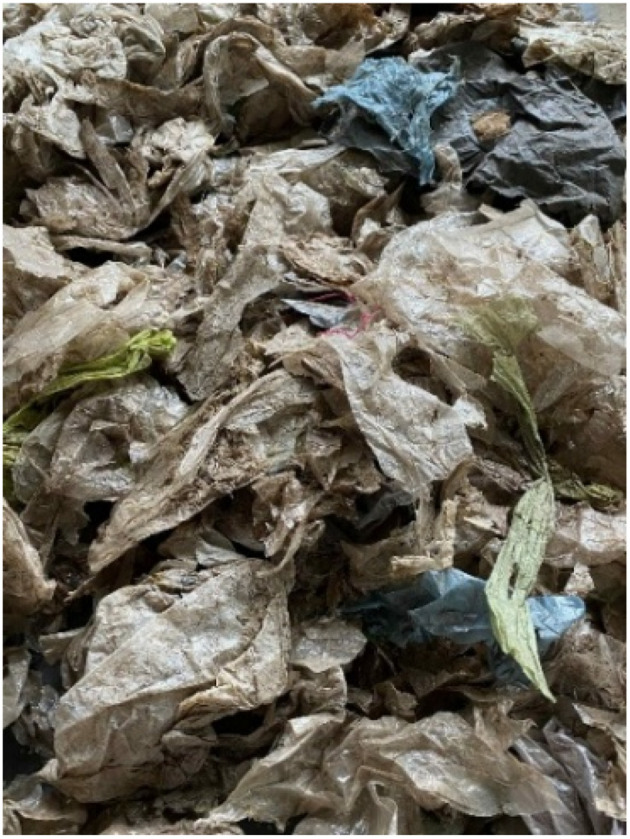
Plastic waste from Hat Yai Municipal landfill.

In response to the growing burden of aged waste and limited landfill space, Hat Yai Municipality has recently initiated landfill excavation and reclamation efforts—commonly referred to as landfill mining. These initiatives aim to recover valuable materials such as plastics, metals, and inert fractions, reduce the volume of buried waste, and reclaim land for future use. The excavation process typically involves mechanical digging using backhoes or loaders, followed by on-site screening with mobile trommel screens. The waste plastic obtained from the screening is shown in Fig. 1.

## 3. Materials

**Fig. 2:**
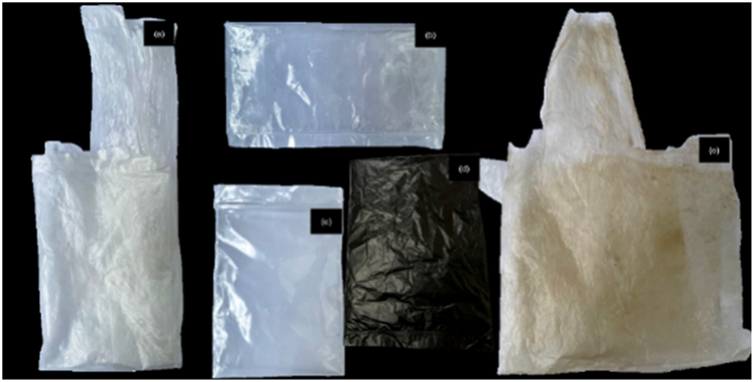
Plastic samples for characterization: (a) PE, (b) PP, (c) PET, (d) PVC, and (e) PE (LF).

This study investigates the properties of two categories of plastic materials: (1) virgin, unused plastics—namely polyethylene (PE), polypropylene (PP), polyethylene terephthalate (PET), and polyvinyl chloride (PVC); and (2) landfill-derived polyethylene [PE (LF)] recovered from municipal solid waste in Songkhla province, Thailand. Landfill-aged PE (LF) was selected as a representative post-consumer plastic from the Hat Yai Municipality landfill, as polyethylene constitutes the dominant fraction of plastic waste found at the site.^35^ These materials were selected to examine changes in their mechanical, physical, thermal, and chemical properties due to long-term environmental exposure. The study focuses on their potential for future research in waste-to-energy conversion, offering a promising approach for sustainable waste management and energy recovery. All plastic samples were thoroughly cleaned, air-dried, and cut into test specimens for comprehensive analysis. The appearance of the plastic samples was shown in Fig. 2.

## 4. Sample preparation

### 4.1. Surface morphology examination

Surface morphology was analyzed using a high-resolution scanning electron microscope (SEM) to examine the distribution of various components. The samples were cut into small pieces and mounted on aluminum stubs using double-sided carbon tape. Prior to imaging, a conductive layer of gold/palladium (Au/Pd) alloy was applied using a Quorum Technologies SC7620 Mini Sputter Coater. The coating material consisted of gold (Au) and palladium (Pd), and the sputtering system was equipped with a single vacuum pump operating at a flow rate of 50 liters per minute. The sputtering was performed at a current of 20 mA for 60 seconds, resulting in a conductive film approximately 10 nm in thickness. This conductive coating was essential to minimize charging effects during SEM observation and to ensure high-contrast imaging, particularly for non-conductive plastic materials. All SEM analyses were conducted in the Scanning Electron Microscope Laboratory.

### 4.2. Analysis of chemical compositions

Chemical composition analysis was carried out to identify the chemical components of the prepared powder sample. Energy-dispersive X-ray spectroscopy (EDX) has been used as an analytical method to distinguish the characteristic X-ray signals of various elements using energy spectrum analysis. Analysis was further refined using advanced software to process the signals obtained and precisely identify the components of the test samples.

### 4.3. Thermogravimetric analysis (DTG and TGA) testing

Thermal analysis (TGA) and differential thermogravimetry (DTG) are used to study the weight changes of heating materials. The TGA measured the weight of the sample at wide temperatures ranging from 30 °C to 800 °C, while the DTG provided detailed information on the weight loss rate. This comprehensive analysis provides information on weight loss, decomposition and chemical reactions occurring during heating processes and helps to understand the behavior of materials during thermal degradation.

### 4.4. Tensile test

**Fig. 3:**
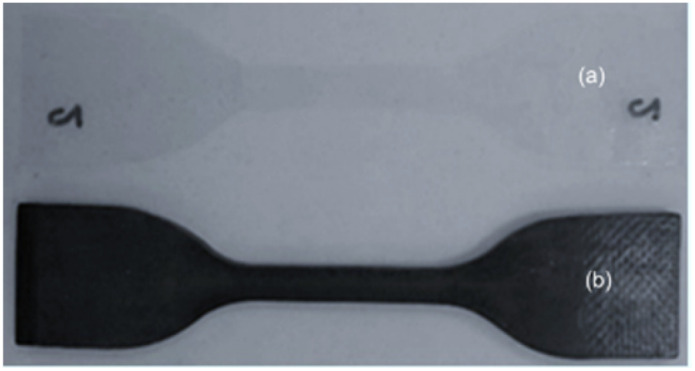
Tensile test specimens: (a) PE and (b) PVC.

Tensile testing is a crucial procedure used in this research to evaluate the mechanical properties of plastic materials, following the ASTM D412 standard. Dumbbell-shaped specimens, as shown in Fig. 3, were prepared and tested using a universal testing machine (UTM), specifically the INSTRON 5565 model equipped with a 50 kg load cell. The tests were performed at room temperature to determine key parameters, including tensile strength (the maximum stress a material can endure while being stretched), Young's modulus (the stiffness of the material), and percentage elongation at break (% Elongation). The testing conditions were strictly controlled with a distance between grips of 115 mm, a gauge length of 25 mm, and a testing speed of 500 ± 50 mm min^−1^. For each sample type, three specimens were tested to ensure statistical relevance. The obtained data are essential for assessing the mechanical performance, flexibility, and potential degradation behavior of the materials under investigation.

### 4.5. Fourier Transform Infrared (FTIR) spectroscopy test

Fourier Transform Infrared (FTIR) spectroscopy is employed to analyze the functional groups and chemical bonds of plastic samples. FTIR provides a spectrum that reveals the infrared absorption of various molecular groups in the materials. This technique enables the identification of chemical structures and functional groups, offering insights into chemical changes that may occur during the landfilling and degradation processes of plastics.

The advanced analyses such as molecular weight distribution, and crystallinity changes are planned for future investigation.

## 5. Results and discussion

### 5.1. Surface morphology (SEM)

**Fig. 4:**
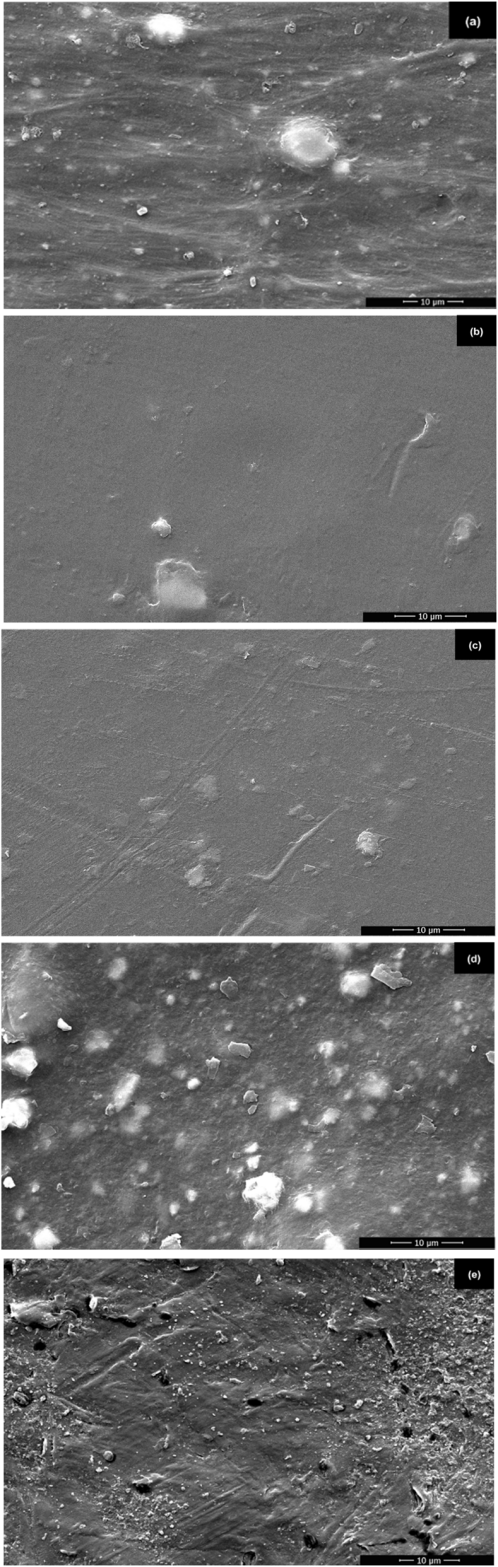
SEM micrographs: (a) PE, (b) PP, (c) PET, (d) PVC, (e) PE (LF).

Fig. 4 shows scanning electron microscopy (SEM) images of four types of commercial polyethylene (PE), polypropylene (PP), polyethylene terephthalate (PET), polyvinyl chloride (PVC), and landfill-derived polyethylene [PE (LF)] from a municipal landfill in Songkhla province, Thailand. Each polymer exhibits distinct surface morphological characteristics reflective of its material type, processing method, and environmental exposure history. The commercial PE sample (Fig. 4(a)) shows a relatively smooth surface morphology, interspersed with small spherical particulates, which may be residues of additives or crystallised polymer domains. The PP sample (Fig. 4(b)) shows the smoothest and most uniform surface among all samples, with minimal striations or surface irregularities, indicating excellent melt flow and homogeneity achieved during processing. This surface uniformity is consistent with the semicrystalline nature and high processability of PP. The PET sample (Fig. 4(c)) exhibits a moderately smooth surface with visibly aligned grooves, likely the result of molecular orientation during extrusion. The PVC sample (Fig. 4(d)) presents a markedly rougher surface texture with scattered irregularly shaped particles. Such features may result from phase separation, incomplete mixing of plasticisers or fillers, or early-stage thermal degradation during processing. Finally, the PE sample recovered from landfill [PE (LF)] (Fig. 4(e)) exhibits the most severely degraded surface, with prominent cracks, rough and uneven textures, fibrous residues, and embedded contaminants.

### 5.2. Elemental composition (EDX)

**Fig. 5:**
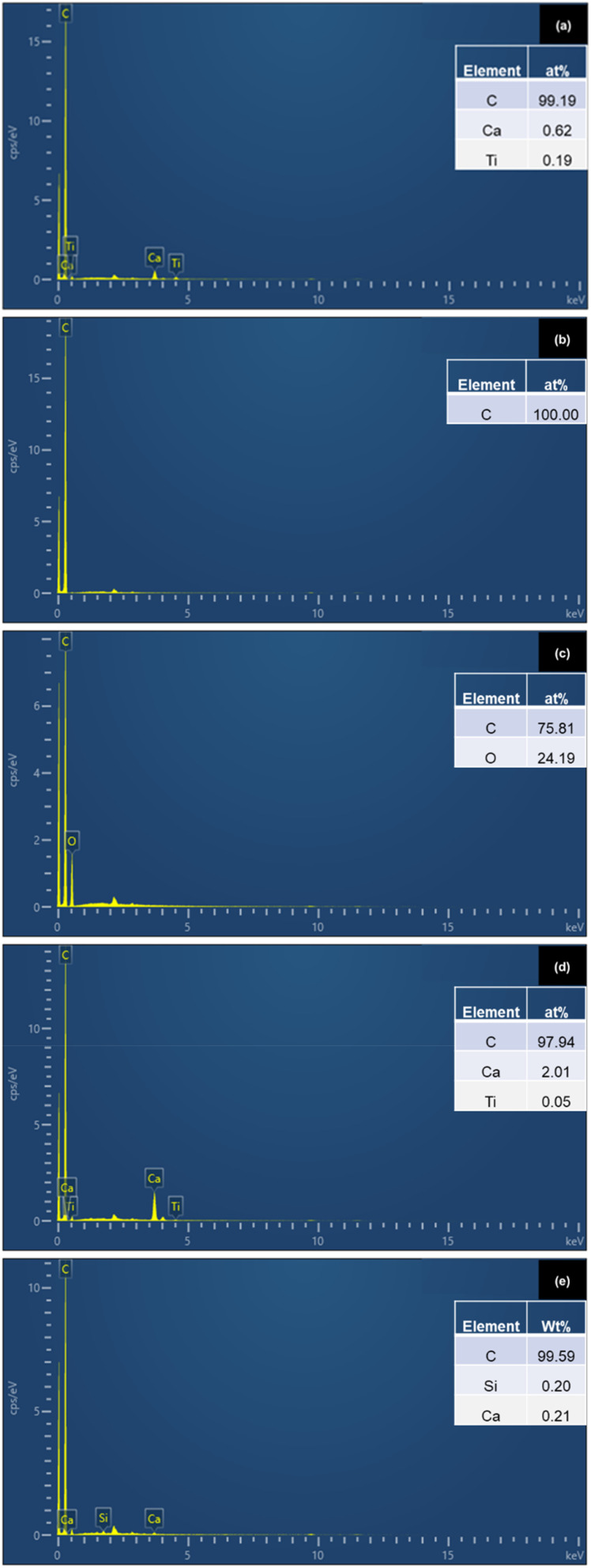
EDX spectra: (a) PE, (b) PP, (c) PET, (d) PVC, (e) PE (LF).

Fig. 5 presents the surface elemental composition (in atomic percentage) of five types of plastics: commercial polyethylene (PE), polypropylene (PP), polyethylene terephthalate (PET), polyvinyl chloride (PVC), and landfill-derived polyethylene [PE (LF)] from a municipal landfill in Songkhla province, Thailand. This analysis aimed to investigate the distribution of elements and detect possible contaminants or degradation products on plastic surfaces.

The commercial PE sample (Fig. 5(a)) consisted mainly of carbon (99.19 at%), with small amounts of calcium (0.62 at%) and titanium (0.19 at%), suggesting a relatively pure polymer structure with minor inorganic additives likely introduced during manufacturing. The PP sample (Fig. 5(b)) contained 100% carbon, indicating high chemical purity and the absence of detectable inorganic elements. The PET sample (Fig. 5(c)) exhibited a lower carbon content (75.81 at%) and a significant amount of oxygen (24.19 at%), which is consistent with its polar ester groups. PVC (Fig. 5(d)) showed carbon as the major element (97.94 at%), along with calcium (2.01 at%) and titanium (0.05 at%). These elements are commonly found in PVC due to the addition of calcium carbonate (CaCO_3_) as a filler and calcium stearate as a thermal stabilizer, both of which enhance mechanical strength and heat resistance. Titanium dioxide (TiO_2_) is another common additive, used as a white pigment and UV stabilizer to prevent photo-degradation and maintain surface appearance. These additive functions are well-documented in commercial and research-grade plastics, as confirmed by Cuthbertson *et al.*,^36^ who found both Ca and Ti to be among the most common additives in PE and PVC formulations.

The landfill-derived PE (LF) sample (Fig. 5(e)), carbon was again dominant (99.59 at%), with trace levels of silicon (0.20 at%) and calcium (0.21 at%). The presence of silicon in this case is likely not from manufacturing but rather from environmental contamination. Prolonged exposure to sunlight, heat, moisture, and microbial activity in landfill conditions promotes surface oxidation of the plastic. This leads to the formation of oxygen-containing functional groups (*e.g.*, carbonyl, carboxyl, hydroxyl), which have a higher tendency to bind with metal ions or inorganic particles such as silica present in the surrounding soil or leachate. Consequently, silicon may adsorb onto the oxidized plastic surface, particularly in low-crystallinity regions where surface interaction is enhanced.

### 5.3. Thermal stability (TGA/DTG)

This study investigates the property of commercial polyethylene (PE), polypropylene (PP), polyethylene terephthalate (PET), polyvinyl chloride (PVC), and landfill-derived polyethylene [PE (LF)] from a municipal landfill in Songkhla province, Thailand. The analysis was performed using thermogravimetric analysis (TGA) and derivative thermogravimetric analysis (DTG) to assess the thermal stability, degradation patterns, and potential for energy recovery of these materials. The results provide insights into the performance of these plastics, focusing on their suitability for thermochemical recycling processes such as pyrolysis, which could convert waste plastics into valuable energy sources.

**Fig. 6:**
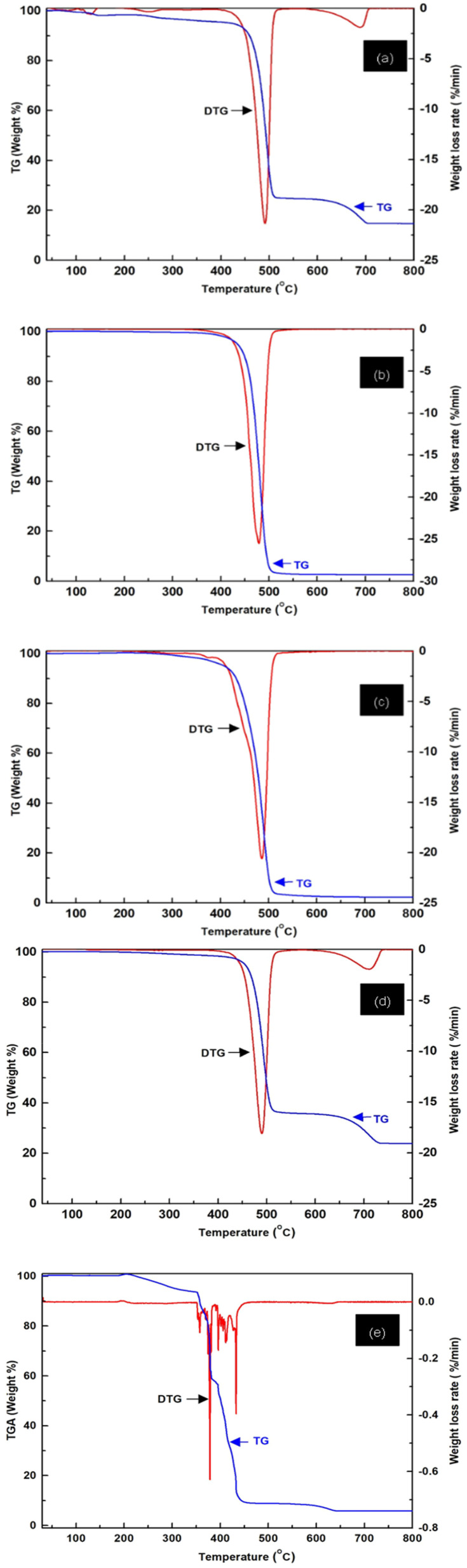
TG/DTG analysis: (a) PE, (b) PP, (c) PET, (d) PVC, (e) PE (LF).

Polyethylene (PE), one of the most widely used commercial plastics, exhibited an initial weight loss of around 103 °C due to moisture evaporation, shown in Fig. 6(a). This was followed by a significant degradation phase at 229 °C, where the polymer backbone began to break down. At higher temperatures, the main mechanism is free radical chain scission, where C–C bonds in the polymer backbone are cleaved, leading to the formation of smaller hydrocarbon molecules and volatiles. The degradation continued at a higher temperature of 469 °C, resulting in a final mass retention of 23% at 650 °C. These findings suggest that PE undergoes a relatively efficient decomposition process, with a significant portion of its mass being converted into gaseous products, making it suitable for energy recovery *via* pyrolysis. The rapid release of hydrocarbons observed in this study is consistent with previous research by Tarani *et al.* (2024),^37^ which highlighted the potential of PE as a fuel during thermochemical conversion.

Fig. 6(b) of polypropylene (PP), degradation pattern to that of PE. The material experienced an initial weight loss at 158 °C, likely due to moisture and low-molecular weight additives, followed by a pronounced weight loss phase at 455 °C, where the polymer structure started to degrade significantly. Like PE, PP degrades mainly through random main-chain scission at high temperatures, producing volatile hydrocarbons and smaller fragments.

This mechanism ensures efficient and rapid decomposition, which is advantageous for energy recovery processes such as combustion or pyrolysis. PP undergoes rapid decomposition, releasing hydrocarbons efficiently, making it an ideal candidate for energy generation by combustion or further fuel production, as supported by the findings of Majder-Lopatka *et al.*^38^

Polyethylene terephthalate (PET), as shown in Fig. 6(c), is widely used in beverages and textile fibres, with a distinct thermal degradation behaviour. The initial moisture evaporation was detected at 231 °C, followed by the onset of a large thermal degradation at 353 °C, and a subsequent phase of degradation occurred at 421 °C. The main chemical mechanism for PET is the cleavage of ester bonds, which leads to the formation of carboxylic acid-terminated and vinyl-terminated chains. In the presence of moisture, hydrolysis can also occur, further breaking down the polymer. A final mass retention of 56% was observed at 473 °C. The high calorific value of PET and the thermal stability position it as a promising candidate for waste-to-energy applications, with pyrolysis and gasification being particularly suitable methods for energy recovery. These results are consistent with previous studies by Majder-Lopatka *et al.*,^38^ which discussed the potential of PET in thermochemical recycling and energy generation.

Polyvinyl chloride (PVC) displayed a different degradation behavior, with initial decomposition observed at 189 °C and significant weight loss occurring at 468 °C, as presented in Fig. 6(d). The primary chemical mechanism for PVC is dehydrochlorination, where HCl is released from the polymer chain, leaving behind polyene sequences. This process can lead to the formation of toxic by-products and requires careful management to minimize environmental and health risks. The final mass storage at 670 °C was 35%. Although PVC can theoretically be converted into energy, the impact of its degradation on the environment must be carefully considered. Thermal degradation of PVC can release harmful by-products, such as hydrogen chloride (HCl) and other toxic compounds, resulting in significant environmental and health risks. Therefore, PVC requires advanced management strategies, including control of pyrolysis or thermal cracking technology, to minimise the release of hazardous substances, as Ledniowska *et al.* pointed out.^39^

In addition to commercial plastics, the study also investigated polyethylene (PE) recovered from a municipal landfill in Songkhla province, Thailand, as shown in Fig. 6(e). Thermogravimetric and derivative thermogravimetric analyses revealed a multistep thermal degradation process, with six major weight-loss events observed at temperatures ranging from 247 °C to 603 °C. These degradation steps corresponded to the loss of volatile compounds, low-molecular weight additives, and progressive breakdown of the polymer backbone. The presence of contaminants and impurities in landfill-derived PE can act as catalysts for oxidative reactions and chain scission, accelerating the degradation process and increasing the yield of volatile products. The total mass loss for PE derived from landfill was approximately 74%, significantly higher than the mass retention of 23% observed for PE virgin at 650 °C. The higher degradation observed in landfill-derived PE can be attributed to the presence of contaminants and impurities accumulated over time in the landfill environment. Despite these impurities, landfill-derived PE retains much of its energy conversion potential. The thermal properties of landfill-derived PE suggest that it can be efficiently converted into hydrocarbon fuels with rapid release, similar to those of virgin PE. This makes landfill-derived PE a promising material for pyrolysis or other thermochemical processes aimed at energy recovery. The use of landfill-derived polyethylene for energy recovery presents an opportunity to repurpose waste materials for sustainable energy production, contributing to both waste reduction and energy generation.

A comparison of landfill-derived polyethylene (PE (LF)) and virgin polyethylene (PE) revealed striking similarities in their thermal degradation profiles. Both materials exhibited moisture evaporation around 103 °C, followed by significant degradation and decomposition. However, PE derived from landfills exhibited a higher degree of degradation, with six distinct weight loss events observed between 247 °C and 603 °C. The total mass loss for PE (LF) was approximately 74%, compared to the mass retention of 23% of virgin PE at 650 °C. This higher degradation in landfill-derived PE is likely due to the accumulation of contaminants from the landfill environment, including dirt, organic matter, and other waste materials. Despite the increased degradation, landfill-derived PE still shows significant potential for energy recovery. The thermal properties of PE (LF) suggest that it can be processed into hydrocarbon fuels with rapid release, similar to virgin PE, making it suitable for energy recovery processes such as pyrolysis. The use of landfill-derived polyethylene for energy recovery presents an opportunity to repurpose waste materials for sustainable energy production, contributing to both waste reduction and energy generation.

### 5.4. Mechanical properties (tensile test)

The tensile performance of various types of plastics has been investigated to assess their mechanical behavior, as shown in Table 1. Polyethylene terephthalate (PET) exhibited the highest tensile strength, with an average value of 106.0 ± 5.9 MPa, demonstrating its superior mechanical integrity. This is consistent with Horvath *et al.*,^40^ who reported that PET is ideal for applications requiring high tensile strength and dimensional stability. In contrast, polyvinyl chloride (PVC) displayed the lowest tensile strength at 3.0 ± 4.9 MPa, reflecting its high flexibility and suitability for non-load-bearing applications. Polyethylene (PE) demonstrated a moderate tensile strength of 74.0 ± 12.0 MPa, while polypropylene (PP) showed a lower tensile strength of 24.0 ± 19.7 MPa. In terms of elongation at break, PP exhibited exceptional ductility with a maximum elongation of 1255.0 ± 257.3%, making it ideal for applications requiring high flexibility, such as packaging films and plastic bags. Conversely, PET had the lowest elongation at break of 53.0 ± 10.0%, indicating brittleness and limited stretchability. PE offered a balanced performance with an elongation of 130.0 ± 59.6%, suggesting its potential for a broad range of applications where moderate strength and flexibility are required. Young's modulus further illustrated the stiffness of the materials, with PET again showing the highest value at 5.26 ± 2.80 N m^−2^, confirming its resistance to deformation under applied stress. PVC, in contrast, had the lowest Young's modulus at 0.26 ± 0.20 N m^−2^, consistent with its flexible nature. A comparative analysis between virgin PE and PE recovered from landfill sources highlighted the mechanical degradation due to environmental exposure. The tensile strength of landfilled PE decreased from 74.0 ± 12.0 MPa to 36.2 ± 2.8 MPa, while Young's modulus dropped from 3.84 ± 0.50 N m^−2^ to 1.19 ± 0.60 N m^−2^. These reductions suggest chemical and structural changes, such as oxidation and polymer chain scission, likely induced by prolonged exposure to landfill conditions. The results align with Canopoli *et al.*,^41^ who observed that buried plastics degrade under anaerobic and moist environments, resulting in diminished mechanical properties. Despite the observed deterioration, PE recovered from landfills retains characteristics that may be applicable in low-performance applications. It also holds potential for use in waste-to-energy processes, where plastic waste can be thermally converted into heat and subsequently electricity. Understanding the mechanical profile of such degraded plastics is crucial for evaluating their feasibility in recycling and energy recovery initiatives. These findings underscore the importance of integrating post-consumer plastic waste into sustainable material management and energy strategies.

**Table 1:** Mechanical properties of selected plastics^a^

Plastic type	Tensile strength (MPa)	Elongation (%)	Young's modulus (N m^−2^)
PE	74.0 ± 12.0	130.0 ± 59.6	3.84 ± 0.50
PP	24.0 ± 19.7	1255.0 ± 257.3	1.26 ± 0.40
PET	106.0 ± 5.9	53.0 ± 10.0	5.26 ± 2.80
PVC	3.0 ± 4.9	329.0 ± 16.3	0.26 ± 0.20
PE (LF)	36.2 ± 2.8	70.5 ± 45.5	1.19 ± 0.60

a LF = landfilled sample collected from Songkhla, Thailand.

Polyethylene recovered from a landfill site in Songkhla province [PE (LF)] was selected for FTIR analysis due to its visibly degraded morphology, elemental contamination, and altered thermal and mechanical properties. SEM images revealed severe surface deterioration, including cracks and fibrous residues, indicative of long-term environmental exposure. EDX analysis confirmed the presence of oxygen and calcium, suggesting oxidative degradation. TGA/DTG results showed multiple degradation steps and higher mass loss compared to virgin PE, confirming chemical transformation. Moreover, tensile testing demonstrated significant mechanical deterioration, reflecting polymer chain scission. FTIR analysis, therefore, serves to confirm the presence of oxidized functional groups and degradation byproducts. This insight supports the use of PE (LF) as a candidate for fuel recovery *via* pyrolysis, owing to its enhanced thermal reactivity and remaining calorific potential.

### 5.5. Chemical structure (FTIR)

Plastic waste contributes significantly to environmental burdens due to its resistance to degradation and long-term persistence. Among various characterization tools, Fourier Transform Infrared spectroscopy (FTIR) is widely used for identifying functional groups and assessing polymer degradation levels, especially in post-consumer waste.

**Fig. 7:**
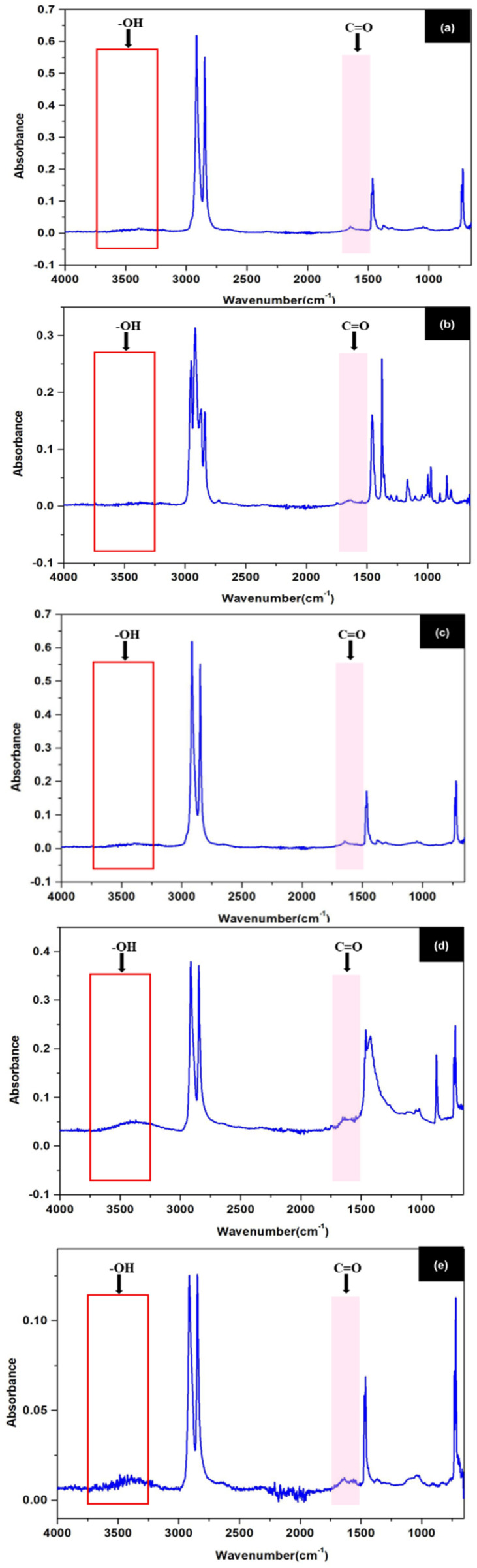
FTIR analysis: (a) PE, (b) PP, (c) PET, (d) PVC, (e) PE (LF).

Polyethylene (PE) exhibits strong CH_2_ stretching bands at 2850–2950 cm^−1^ and bending/rocking modes around 1470 cm^−1^ and 720 cm^−1^, characteristic of semicrystalline polyethylene,^42^ as shown in Fig. 7(a). The absence of absorbance in the 3200–3600 cm^−1^ and near 1700 cm^−1^ regions indicates negligible hydroxyl and carbonyl content, confirming that the material remains chemically intact and is suitable for mechanical recycling without prior treatment.^43^

The FTIR spectrum of PET features a strong carbonyl (C=O) peak between 1720–1740 cm^−1^, linked to its ester backbone, and prominent C–O stretching between 1250–1100 cm^−1^. Aromatic character is confirmed by C–H stretches at 3000–3100 cm^−1^ and out-of-plane bending near 870–750 cm^−1^,^42^ as shown in Fig. 7(b). No degradation peaks (OH or additional C=O groups) are observed, implying structural preservation. As such, PET is more viable for material recycling than energy recovery, given its thermal stability and integrity.^44^

The FTIR profile of PP exhibits strong CH_2_ and CH_3_ stretching bands at 2850–2950 cm^−1^ and bending modes between 1370–1450 cm^−1^, consistent with isotactic polypropylene,^42^ as shown in Fig. 7(c). Absence of oxidation-related bands such as –OH (3200–3600 cm^−1^) or C=O (∼1700 cm^−1^) indicates that the polymer has not undergone environmental degradation. The chemical inertness of this sample suggests dual usability—for both mechanical reuse and fuel conversion, with minimal pretreatment.^45^

PVC's FTIR spectrum contains CH stretching bands around 2850–2950 cm^−1^ and a prominent C–Cl stretching peak near 690 cm^−1^, a unique diagnostic for this polymer,^42^ as shown in Fig. 7(d). Minimal or absent carbonyl peaks (∼1710 cm^−1^) and hydroxyl groups suggest limited oxidative degradation. However, despite its stability, the presence of chlorine atoms poses serious environmental and health risks during thermal processing. Thus, while fuel recovery is possible, it requires dechlorination or highly controlled pyrolysis conditions.^46^

The FTIR spectrum of PE (LF) shows key functional groups associated with the degradation of polymers. As shown in the experimental results in Fig. 7(e), a large band in the region of 3200–3600 cm^−1^ corresponds to OH stretching vibrations, and a distinct carbonyl (C=O) peak near 1700–1750 cm^−1^, both absent in virgin PE. These characteristics are typical of oxidative degradation processes occurring in polyethylene during long-term environmental exposure, including photooxidation and thermal oxidation. The presence of oxygen-containing functional groups, such as hydroxyl and carbonyl, indicates a degree of polymer oxidation that can reduce molecular weight and increase thermal reactivity. These chemical changes are beneficial for thermochemical conversion processes such as pyrolysis. Oxidized polymers generally require lower activation energy for thermal decomposition and are therefore better suited as fuel. Furthermore, it has been reported that degraded polyethylene produces a large amount of hydrocarbons during pyrolysis, which can be refined into liquid fuels or used directly for energy recovery. These conclusions are consistent with the work of Mankhair *et al.*,^47^ which demonstrated that excavated plastic waste from landfills shows significant degradation and retains potential for resource recovery through energy conversion technologies. FTIR evidence supports the idea that PE (LF), despite environmental ageing, retains its heat value and can serve as a viable alternative energy source, contributing to the objectives of the circular economy and reducing dependence on fossil fuels.

To confirm the suitability of landfill-derived PE for energy recovery *via* pyrolysis, Koti *et al.*^35^ investigated the pyrolysis of plastic waste collected from the Hat Yai Municipality landfill. The study focused on mixed plastic waste predominantly composed of PE (approximately 70 wt%). Under optimum catalytic and operating conditions, the maximum yield of pyrolysis oil reached approximately 47 wt%, and the resulting oil exhibited high quality with a higher heating value (HHV) of around 38 MJ kg^−1^. Further compositional analysis of the pyrolysis oil revealed characteristics similar to fossil fuels, comprising gasoline (54.35–56.33 wt%), jet fuel (14.61–15.45 wt%), and diesel (12.57–15.08 wt%). Considering the contaminants, a comparative study between washed and unwashed plastic waste was conducted. The results showed that the presence of contaminants in unwashed plastic waste slightly increased the pyrolysis temperature but significantly reduced the product yield. However, the pyrolysis products from both cases exhibited comparable higher heating values (HHVs). Additional details can be found in the work of Koti *et al.*^35^

## 6. Conclusions

This study provides a comprehensive analysis and multilevel characterisation of five types of plastics: commercial polyethylene (PE), polypropylene (PP), polyethylene terephthalate (PET), polyvinyl chloride (PVC), and landfill-derived polyethylene [PE (LF)] from a municipal landfill in Songkhla province, Thailand. Extended exposure to environmental factors such as moisture, oxygen, and high temperature in landfills was found to cause significant degradation of surface structures, thermal stability, mechanical strength, and chemical composition. SEM analysis revealed significant cracks and surface porosity in PE (LF), contrary to smooth and stable surfaces of commercial plastics. TGA/DTG results indicated that PE (LF) experienced significant mass loss starting around 350 °C, whereas commercial plastics remained thermally stable above 450 °C. Mechanical testing confirmed the reduced performance of PE (LF), with tensile strength less than 10 MPa compared to 25–35 MPa for commercial plastics. Chemical analyses (FTIR, EDX) identified the presence of new functional groups, such as carbonyl (C=O) and hydroxyl (–OH), resulting from oxidative degradation in landfill environments. Despite this degradation, PE (LF) retains considerable potential for conversion to alternative energy through pyrolysis, positioning it as a promising candidate for sustainable energy recovery.

These findings underscore the importance of systematically evaluating landfill plastics for resource recovery and highlight the need for continued research into effective waste management strategies. For practical application, it is recommended that waste management authorities and recycling industries develop targeted landfill mining and separation processes to recover degraded plastics for energy production, thereby reducing landfill burden and supporting the transition to a circular economy. This approach is highly relevant to stakeholders, including local governments, recycling industries, and energy producers, as it offers a pathway to mitigate environmental impacts, optimize resource use, and advance sustainable development goals in regions facing plastic waste challenges.

## Conflicts of interest

There are no conflicts to declare.

## Data Availability

The original data of the study are included in the article. Further inquiries can be directed to the corresponding authors (Email: parinya.kh@psu.ac.th).
